# Giant rhyolite lava dome formation after 7.3 ka supereruption at Kikai caldera, SW Japan

**DOI:** 10.1038/s41598-018-21066-w

**Published:** 2018-02-09

**Authors:** Yoshiyuki Tatsumi, Keiko Suzuki-Kamata, Tetsuo Matsuno, Hiroshi Ichihara, Nobukazu Seama, Koji Kiyosugi, Reina Nakaoka, Kazuo Nakahigashi, Hideaki Takizawa, Kazuki Hayashi, Tatsuro Chiba, Satoshi Shimizu, Mamoru Sano, Hikaru Iwamaru, Haruhisa Morozumi, Hiroko Sugioka, Yojiro Yamamoto

**Affiliations:** 1 Kobe Ocean-Bottom Exploration Center, Kobe University, Kobe, Japan; 2 Earthquake and Volcano Research Center, Nagoya University, Nagoya, Japan; 3 Department of Planetology, Kobe University, Kobe, Japan; 4 Organization of Advanced Science and Technology, Kobe University, Kobe, Japan; 5 Department of Marine Resources and Energy, Tokyo University of Marine Science and Technology, Tokyo, Japan; 6 Johnny & Associates, Tokyo, Japan; 7 Asia Air Survey, Tokyo, Japan; 8 Nippon Marine Enterprises, Yokosuka, Japan; 9 Japan Oil, Gas and Metals National Corporation, Tokyo, Japan; 10 Japan Agency for Marine-Earth Science and Technology, Yokosuka, Japan

## Abstract

Kikai submarine caldera to the south of the Kyushu Island, SW Japan, collapsed at 7.3 ka during the latest supereruption (>500 km^3^ of magma) in the Japanese Archipelago. Multi functional research surveys of the T/S Fukae Maru in this caldera, including multi-beam echosounder mapping, remotely operated vehicle observation, multi-channel seismic reflection survey, and rock sampling by dredging and diving, provided lines of evidence for creation of a giant rhyolite lava dome (~32 km^3^) after the caldera collapse. This dome is still active as water column anomalies accompanied by bubbling from its surface are observed. Chemical characteristics of dome-forming rhyolites akin to those of presently active small volcanic cones are different from those of supereruption. The voluminous post-caldera activity is thus not caused simply by squeezing the remnant of syn-caldera magma but may tap a magma system that has evolved both chemically and physically since the 7.3-ka supereruption.

## Introduction

Supereruptions leading to the huge caldera collapse are rare but extremely hazardous events, and also have severe global impacts such as ‘volcanic winter’^1,2^. Many of these supervolcanoes repeat supereruptions in their multi-million year histories^3–7^. Although the volcanic activity is relatively quiet during the intervening periods between supereruptions, the post-caldera activity should provide a key to understanding the evolution of magma-plumbing system in the whole caldera cycle. The reason for believing so is that dynamic activities in this period such as formation of resurgent domes, i.e., uplift of the caldera floor by extensive magmatic intrusions, and volcanic cones may represent processes of not only calming down from the climactic eruption but also preparation for the next supereruption.

Subduction zones, as well as hotspots^8^ and continental rifts^9^, are sites of supereruption. The Japanese Archipelago is built at convergent plate margins where Pacific and Philippine Sea oceanic plates, are being subducted at trenches and is one of the most densely-distributed volcanic zones on the Earth consisting of 111 active volcanoes with variable eruption styles. At least in Japanese islands, the eruption volume—frequency relationships of caldera-forming supereruption (≥40 km^3^ in dense rock equivalent, DRE) are statistically different from those of smaller summit eruptions, suggesting that different mechanisms control these eruptions^10^. Seven volcanoes prone to supereruptions are built in regions of low crustal strain rate, leading to the speculation that the viscous silicic melts that cause such eruptions can be readily segregated from the partially molten lower crust and form a large magma reservoir in such a tectonic regime^10^. Many of these Japanese supereruptions are followed by formation of rather small post-caldera stratovolcanoes, cones and/or lava domes. Among these supervolcanoes, Kikai caldera is distinctive in that a large dome is present within the caldera^11^. Six samples collected so far from this dome are rhyolites^12,13^, suggesting that this dome could be a lava dome. However, the origin of this dome has been debated due to the lack of comprehensive data set such as high-resolution topographic and seismic reflection data, volcanological observation by a remotely operated vehicle (ROV), and sampling and analyses of dome-forming rocks.

**Figure 1:**
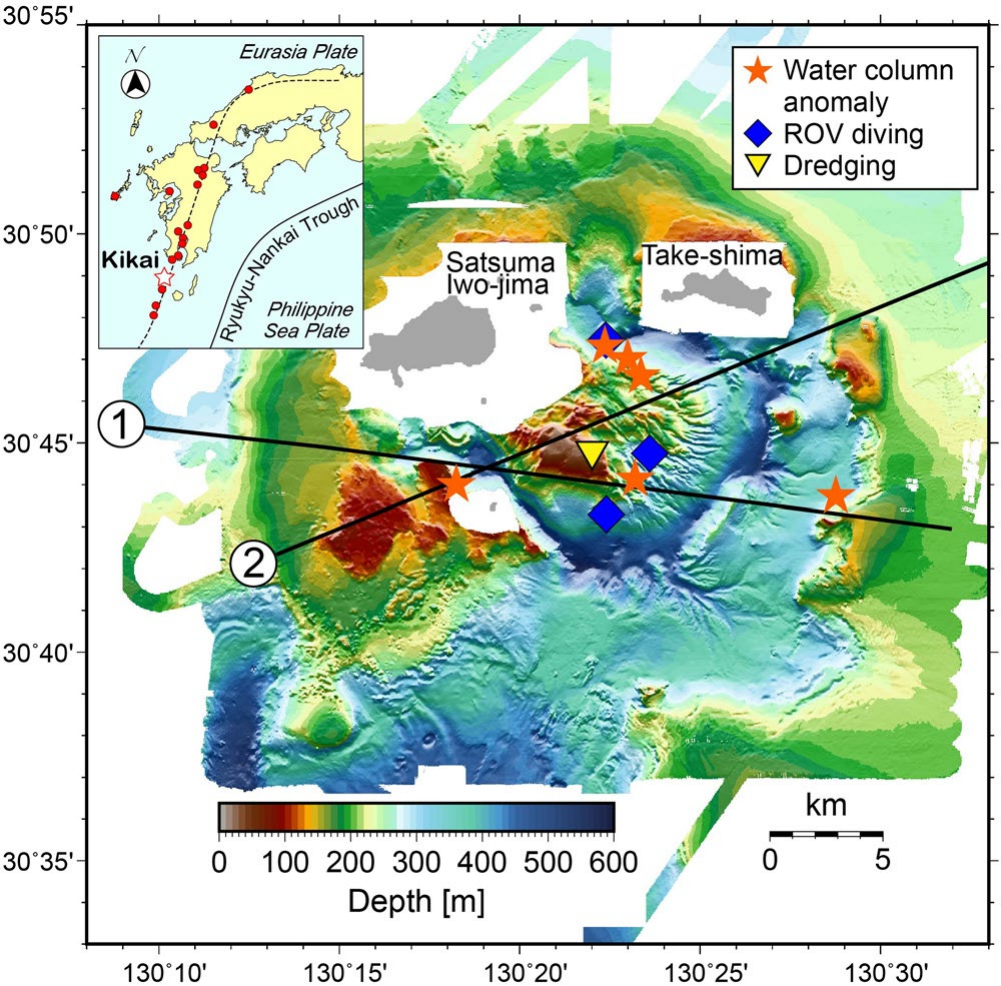
Location and bathymetry of Kikai caldera. This supervolcano forms the volcanic front (broken line) of active volcanoes (red circles) of SW Japan arc. A giant dome is situated on the caldera floor within Kikai caldera. Two MCS lines, distribution of water column anomaly, ROV diving and dredging points are also shown. This figure was produced by using Generic Mapping Tool, GMT, version 5.3.3^27^.

Kikai caldera is located to the south of the Kyushu island and lies astride the volcanic front of the SW Japan arc that is built by subduction of Philippine Sea plate at Nankai trough and Ryukyu trench (Fig. 1). Two islands, Take-shima and Satsuma Iwo-jima, represent subaerial parts of the northern rim of this submarine caldera (Fig. 1).

At least three supereruptions, Koabi, Tozurahama, and Akahoya eruptions at 140, 95 and 7.3 ka with a DRE volume of ~130, 130, and 500 km^3^, respectively, occurred in this volcano^14–17^. The 7.3-ka Akahoya supereruption was commenced by a sequence of plinian pumice-fall and intraplinian pyroclastic flows. This opening-stage activity was followed by the climactic eruption including a voluminous ignimbrite, which spread out across the sea to southern Kyushu Island (>80 km distance), and co-ignimbrite ash, which was dispersed over a wide area of Japanese islands, more than 1000 km far away from this caldera^14,15^. Lines of geological evidence suggest that the latest caldera collapse took place concurrently with the climactic eruption^16^.

Post-caldera volcanic activity^14,18^ was commenced by formation of a rhyolite lava dome at 6 ka followed by growth of a rhyolitic stratovolcano, Iwo-dake, with a total volume of 1.1 km^3^. In addition to these felsic magmatism, a basaltic pyroclastic cone, Inamura-dake (0.1 km^3^) was created at 3.9 ka. The latest activity in this caldera volcano took place in 1934–1935, 2 km east of Satsuma Iwo-jima on 500-m-deep seafloor, with rhyolite lava forming Showa Iwo-jima Island.

## Results

### Caldera structure

**Figure 2:**
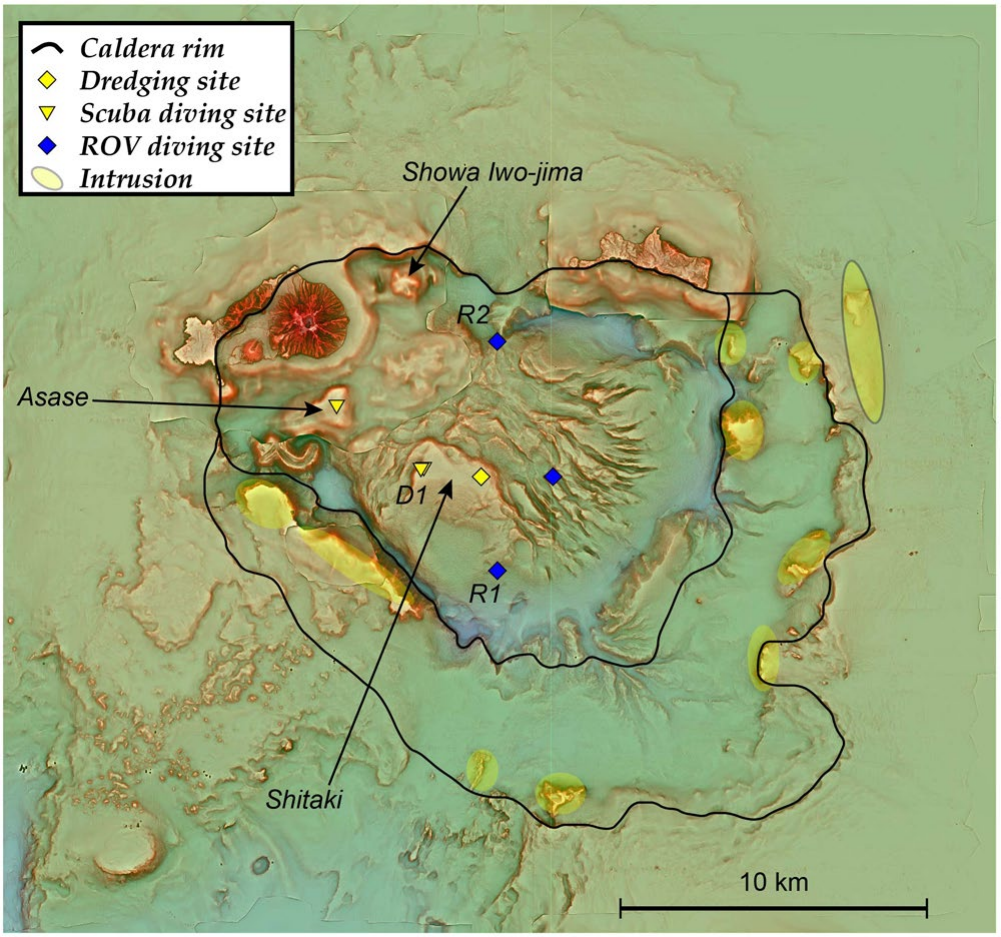
A red relief image map of Kikai caldera based on our bathymetry survey. Topography of the unsurveyed and onland areas is drown by using M-7000 digital data of Japan Hydrological Association and digital elevation model of Geospatial Information Authority of Japan (https://fgd.gsi.go.jp/download/menu.php), respectively. Inner and outer caldera rims are shown by solid lines. Several intrusions (yellow) are distributed along the caldera rims. Dredge (yellow diamond), ROV diving (blue diamonds), and scuba diving (yellow triangles) points are also shown. D1, R1 and R2 are sites where photographs in Figs 4 and 6 were taken. This map was generated using MakeRRIM ver.1.1.0”^28^ that is an Asia Air Survey in-house C program^29^.

**Figure 3:**
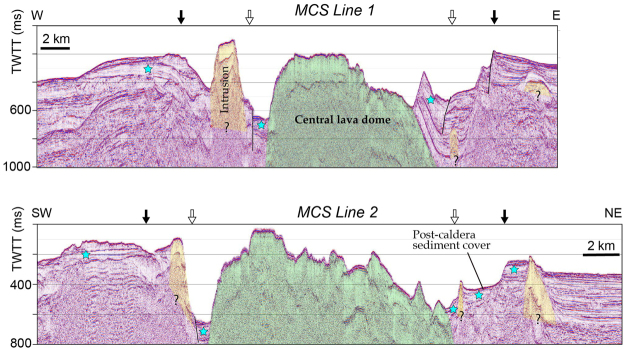
MCS profiles along the lines 1 and 2 (Fig. 1). Topographical and/or structural caldera rims (open and solid arrows for inner and outer rims, respectively), post-caldera sediment cover layers, and possible 7.3-ka syn-caldera ignimbrite layers (blue stars) are highlighted. Central lava dome intruded and upwarped the caldera floor at its margin. Several intrusions are distributed along caldera rims.

A bathymetry map and a red relief image map of Kikai submarine caldera obtained by multi-beam mapping are demonstrated in Figs 1 and 2. It has been suggested that Kikai caldera may exhibit topographically a double-caldera structure^14–16^. This structure, the outer and inner calderas having 24 × 19 and 17 × 15 km (major and minor axes) is confirmed and shown in Fig. 2 based on the location of the topographic depression and normally faulted displacement of subsurface layers documented by the present bathymetry together with the multi-channel seismic (MCS) survey results (Fig. 3). The outer and inner caldera rim may overlap on Satsuma Iwo-jima and Take-shima Islands as previously suggested^16^.

Three supereruptions at 140, 95 and 7.3 ka, took place in this caldera. The following two lines of evidence suggest that the inner caldera may have collapsed by the latest 7.3-ka Akahoya eruption. Firstly, a thin post-caldera sediment layer is underlain by a thick (~30 m) layer likely to be 7.3-ka ignimbrites deposited on both the inside and outside of the caldera (Fig. 3). Secondly, the central dome situated within the inner caldera is not displaced by faults along the inner caldera rim, implying that the doming event took place after, but not caused, the collapse of inner caldera. Although timing of the outer caldera formation is unknown at the present stage, there is no evidence suggesting that the outer caldera collapsed by older supereruptions. Observation that both inner and outer caldera rims overlap along the northern part of this caldera may lead to a speculation that the Kikai double caldera was collapsed by a single supereruption at 7.3 ka.

The volume of caldera collapse for the inner and outer calderas can be estimated to be ~50 and ~90 km^3^, respectively, based on both caldera areas (~170 and ~300 km^2^) and average depths to the caldera floors from the outer caldera edge (~600 and ~300 m). It should be stressed that the total caldera volume (~140 km^3^) is much smaller than the DRE volume of Akahoya supereruption (~500 km^3^).

MCS survey demonstrated the presence of several intrusions or small cones/domes, some appearing above the sea floor (Fig. 3). Although further examination is needed for understanding the relationship between caldera collapses and these intrusions, it should be stressed that these intrusions are distributed along both the inner and outer caldera rims.

### Central dome within the inner caldera

One characteristic topographic feature of Kikai caldera is the existence of domal uplifted body within the inner caldera^11^, which is confirmed and more clearly shown by our high-precision multi-beam mapping (Figs 1 and 2). The dome upheaves from the inner caldera floor (600 m deep) above the sea level to form Asase rock reef (Fig. 2). A small island locating within the caldera is Showa Iwo-jima Island (Fig. 2), which was erupted in 1934–1935 from a vent on the caldera floor to form a lava dome of >300 m height^19^. However, this dome is topographically separated from the major central dome (Fig. 2).

**Figure 4:**
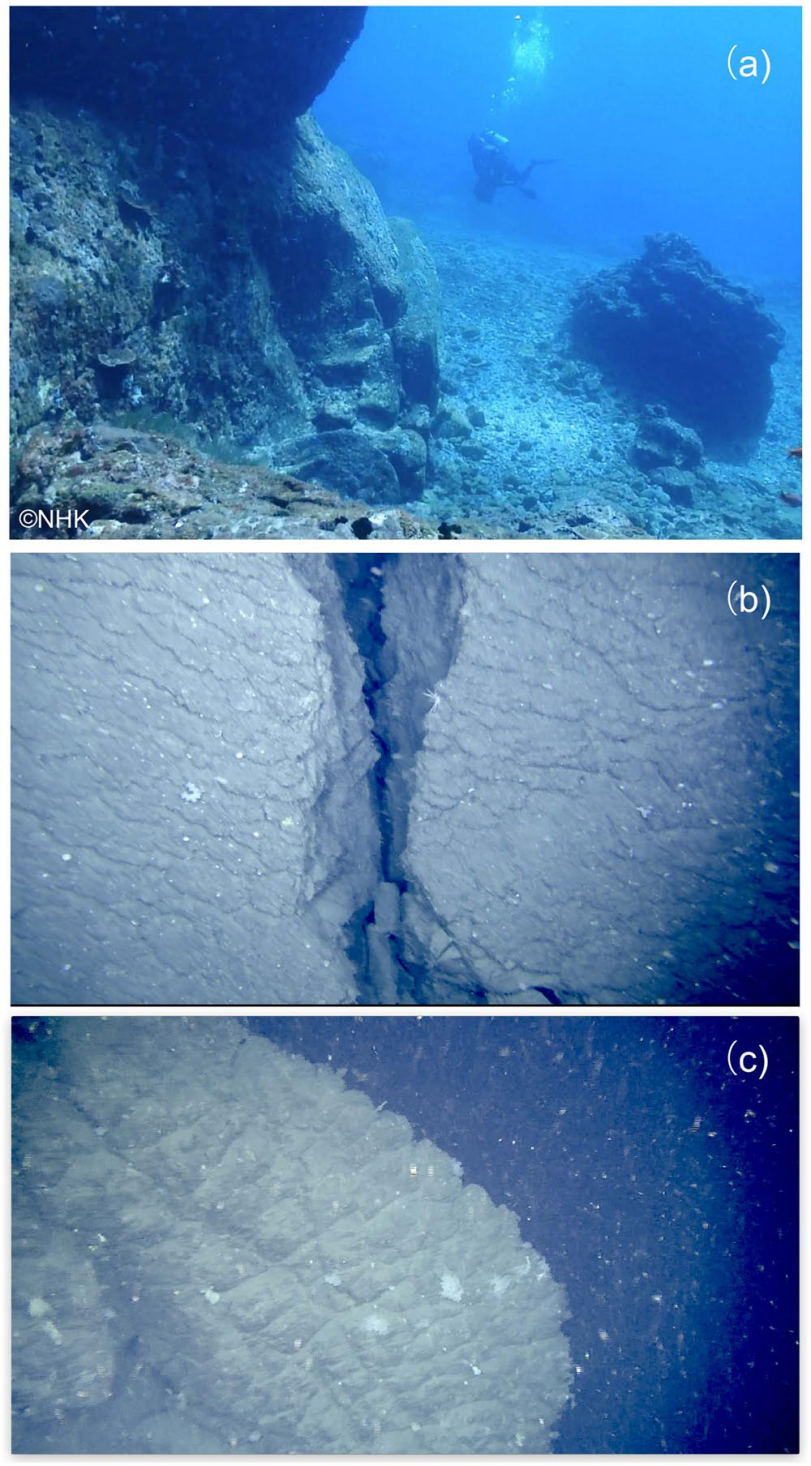
Lavas at the surface of the central dome at the sites D1 (**a**) and R1 (**b**,**c**) in Fig. 2. The surface of lava dome consists of rhyolite blocks with water-chilled, tortoise-shell contraction cracks (**b**) and pillow lobe (**c**) structures. A photograph (**a**) was supplied from NHK.

**Figure 5:**
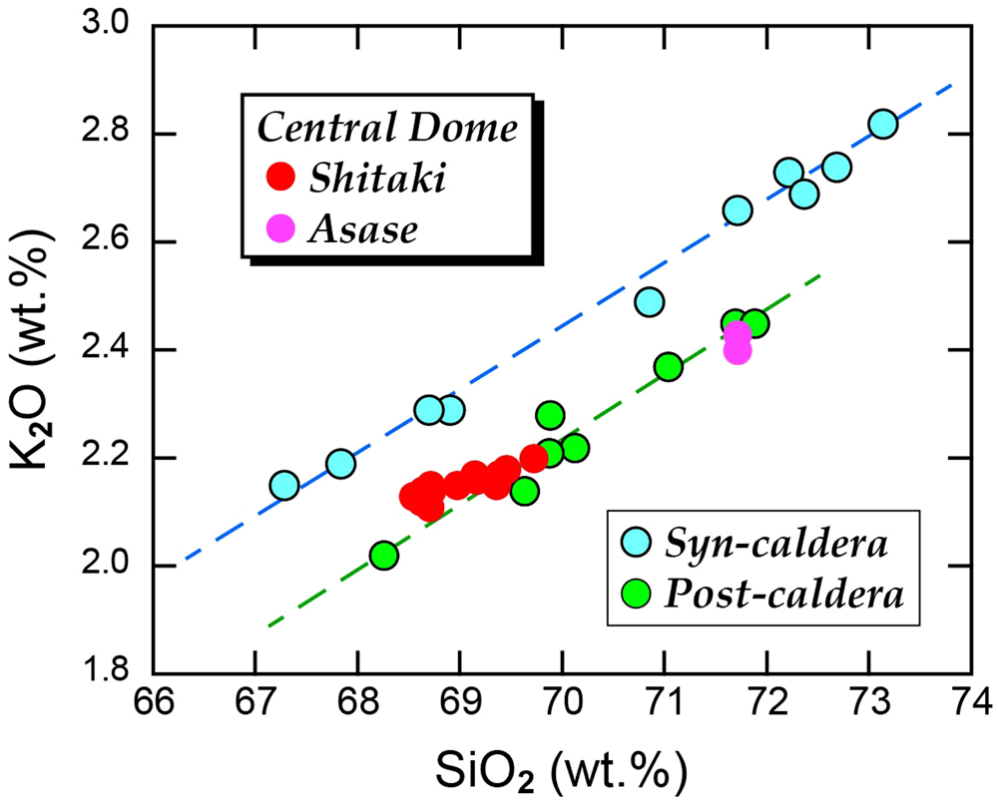
Geochemical characteristics of syn- and post-caldera ejecta, exhibiting two well-defined chemical trends respectively. Rocks recovered from Shitaki and Asase on the central lava dome possess compositions identical to post-caldera rocks. Syn-caldera and central dome rocks were analyzed by our XRF with standard procedures and data for post-caldera rocks are from literatures^14,19^.

The following results suggest that the central high consists of a giant post-caldera lava dome. Firstly, observations by diving at Asase and Shitaki (Fig. 2) and by ROV at three points on the slopes and the margin of the dome (Fig. 2) indicated that the surface of this dome is covered by rhyolite blocks with sizes up to several meters that often exhibit water-chilled structures such as tortoise-shell contraction cracks and in some cases forming pillow lobes (Fig. 4). Secondly, rocks collected by diving and dredging at four points near the peaks of this dome (Fig. 2) are brecciated and vesicular, but not pumiceous, hypersthene-augite rhyolites. These rhyolites exhibit a compositional variation, e.g., 68.5–72 wt.% SiO_2_ (Fig. 5). Such a compositional diversity has been commonly observed for rhyolite lava domes; rhyolites consisting of Showa Iwo-jima (500 × 270 m) show a variation similar to the central lava dome^19^. Thirdly, MCS profiles crossing this mound show that the structural high exhibits a massive and transparent internal structure without any layering and is poorly overlain by the sediment cover (Fig. 3). Diving observations also confirm little sediment cover on the dome surface near the flat-top of Asase and Shitaki (Figs 2 and 4a), excluding the possibility that the dome is a resurgent dome that formed by swelling of the caldera floor with thick pyroclastic sediments^4^. Lastly, the lava dome grows up from the inner-caldera floor that consists of a layer immediately below the post-caldera sediment cover likely to consist of 7.3 ka ignimbrite as typically documented in the western margins of the inner caldera (Fig. 3).

An interesting structure that provides a constraint on the formation age of this central lava dome is observed at the ridge near the southeastern rim of inner caldera. The MCS profile across this ridge (Line 1 in Figs 1 and 3) shows that this ridge is composed of an outward inclined layer that may formally form a caldera floor and is underlain by a massive body having a seismic characteristic identical to the central lava dome. It is thus suggested that this ridge was uplifted by ~300 m along the margin of central dome in consequence of lava dome formation.

Characteristic radial and rift-valley-like surface structures are developed typically on the eastern slope of the dome (Fig. 2). On the other hand, three highlands on the western side exhibit rather smooth surface. There is no difference in the thickness of sediment cover on the smooth and rough surfaces, suggesting that such surface structures may reflect the original topography. This structural difference may provide constraints on the formation process of this lava dome, which will be discussed later.

### Water column anomaly and gas emission

**Figure 6:**
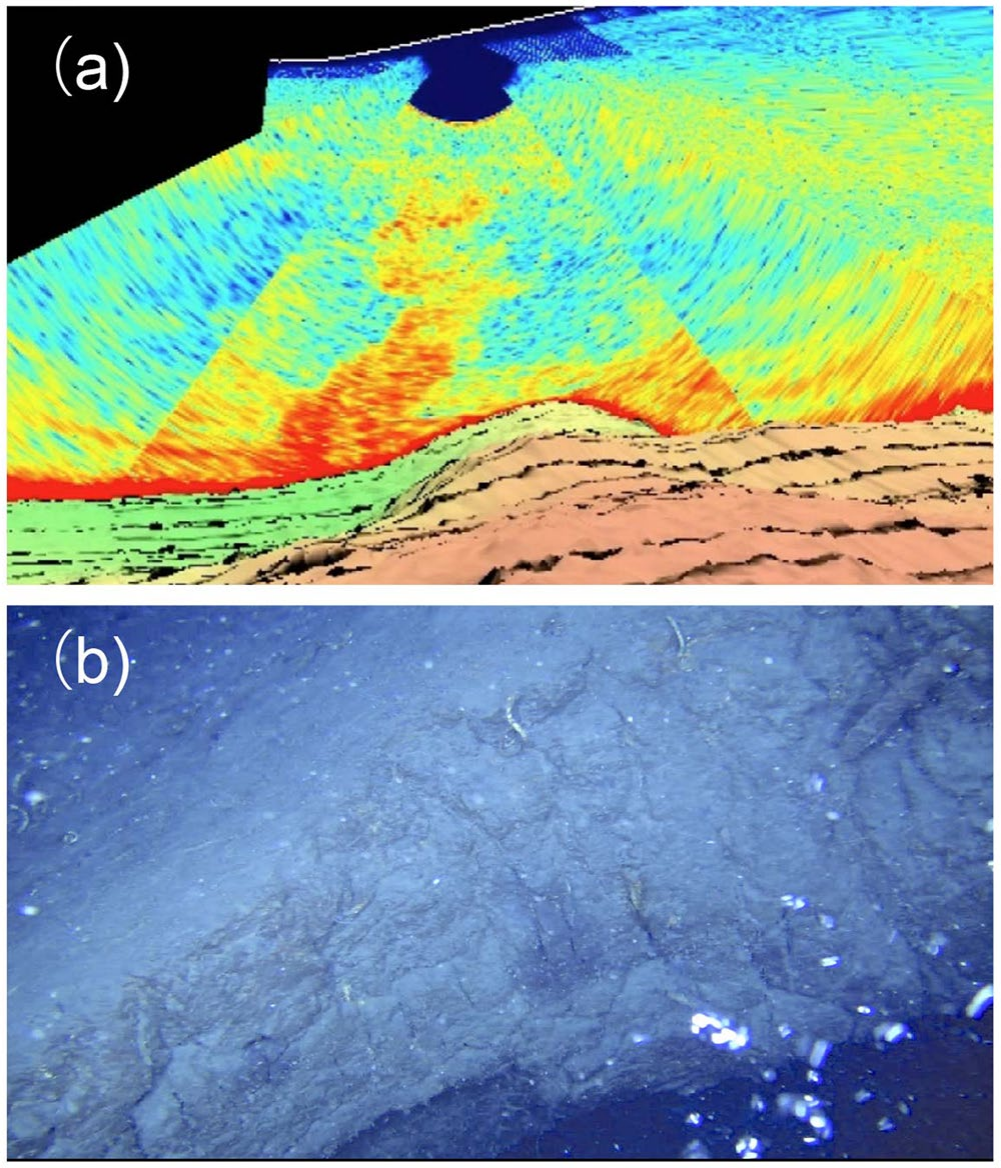
A water column anomaly (**a**) and gas bubbling (**b**) at the dome surface observed at the site R2 in Fig. 2.

Various hydrothermal activities including high (>800 °C) and low temperature fumaroles, hot springs and gas bubbling have been documented in this caldera^20,21^. The major sites of such activities are focused near the summit of a post-caldera volcano, Iwo-dake; Showa Iwo-jima, the latest post-caldera lava dome, is also the site of active gas bubbling. In addition to these, acoustic imaging with a multi-beam echosounder, Kongsberg EM 712 working at 40–100 kHz shows the distribution of acoustic water column anomalies at six points during this survey, four of which are located on the central lava dome (Fig. 1). The largest one forms a >200-m high anomaly above the ~360-m depth seafloor (Fig. 6a). ROV diving at this point confirmed gas emission from the gap of lava blocks (Fig. 6b) although active vents have not been identified.

## Discussion

### Lava dome formation process

Domal structures often emerging at the post-caldera stage of large caldera volcanoes have been known as ‘resurgent domes’^4,22,23^. The resurgent dome is developed after caldera collapse by gradual upwarping of the caldera floor mainly consisting of ignimbrites ejected and deposited by a caldera-forming eruption. It is thus suggested that doming is caused by renewed magma intrusion, not extrusion.

The central dome of Kikai caldera, on the other hand, is composed of subaqueous rhyolite lava and form a giant lava dome on the inner caldera floor with a 10-km diameter, a 600-m height and a ~32-km^3^ volume based on the present survey results. Voluminous post-caldera activity has been reported from Long Valley^3^ and Yellowstone^7^ calderas that were created at a continental rift and a hotspot, respectively. On the other hand, such voluminous post-caldera magmatism is rather rare in subduction zones excepting the Toba resurgent dome, Indonesia^6^. Although lava dome formation is one common post-caldera activity in this tectonic setting, the Kikai central lava dome overwhelms others by its huge volume. The Taupo Volcanic Zone, North Island, New Zealand, is one of the most frequently active and productive Quaternary caldera clusters. Among these, the Okataina Volcanic Centre is characterized by multiple eruptions of rhyolite lava domes after the latest Rotoiti supereruption at 45 ka^24^, such as Haroharo and Tarawera dome complexes. Although the total volume of these lava domes and flows comes up to ~80 km^3^, >20 eruptive vents are recognized, i.e., a ~4 km^3^ outflow in average for each vent; actually, one of the largest lava dome possesses a volume of 2.5 km^3^ ^25^. Creation of lava domes and/or central cones at the post-caldera stage is also common in the Japanese Archipelago. The largest lava dome activity reported so far would be formation of two domes, Naka-jima and Usu-san, (~8 km^3^ in total^18^) after the collapse of Toya caldera in Hokkaido by the 113.5-ka supereruption (~100 km^3^ DRE); one of the largest examples of the post-caldera stratovolcanoes is Naka-dake in Aso caldera, Kyushu, with a DRE volume of ~12 km^3^ ^18^. Formation of a 32-km^3^ post-caldera giant lava dome in Kikai caldera is therefore a noteworthy activity in the caldera cycle.

**Figure 7:**
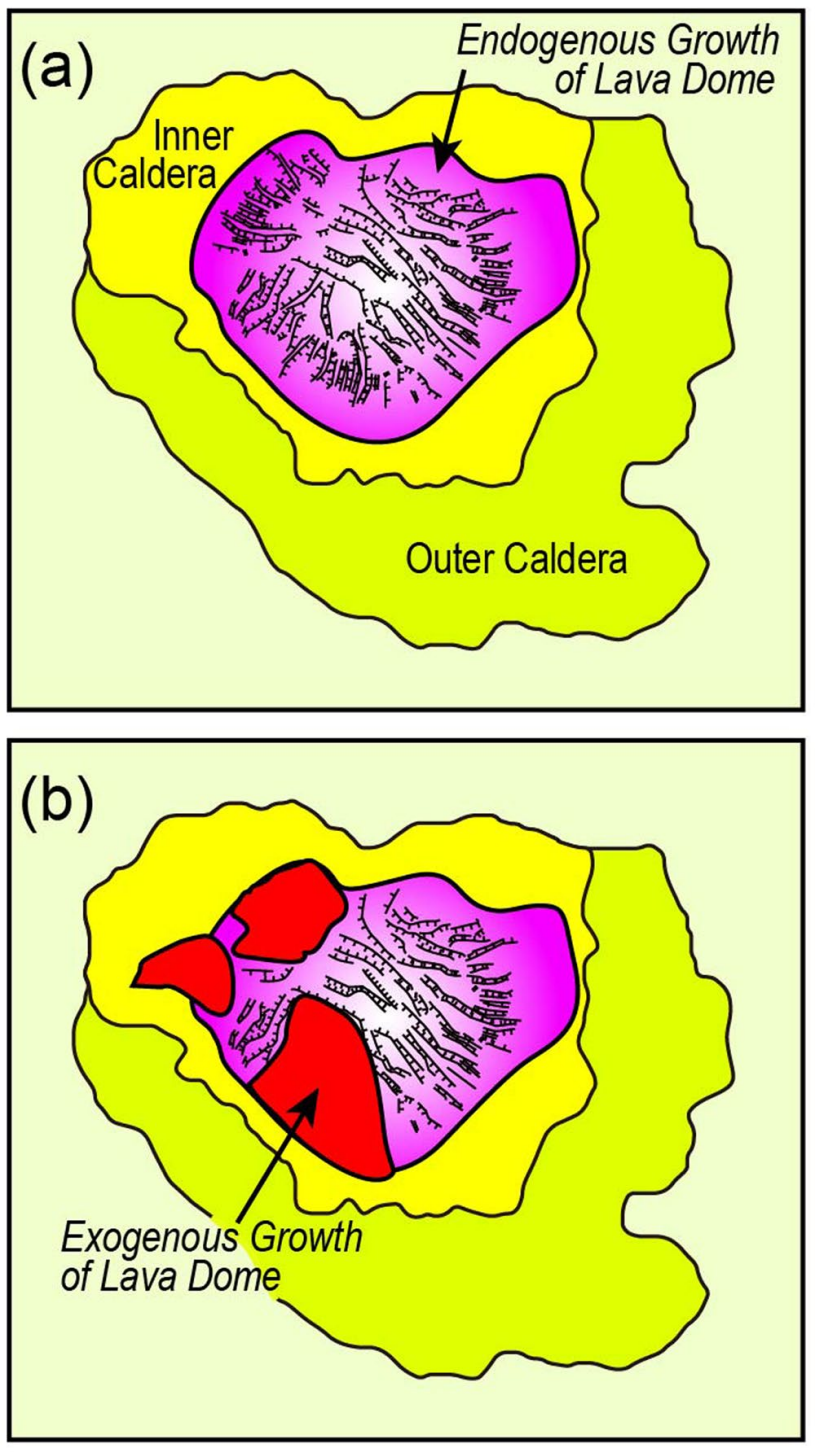
Schematic diagrams showing a possible process of the Kikai lava dome formation. (**a**) The lava dome extruded on flat surfaces and spread laterally and inflate vertically in an endogenous regime, which caused radially developed fractures at its surface. (**b**) Lava flows directly covered the dome surface (exogenous growth) and formed flat surfaces. Valley-like surface structures in (**b**) are traced from Fig. 2.

Although whether this lava dome is monogenetic or composite is not clearly defined solely based on the present data, two distinct topographic features that the Kikai central giant lava dome exhibits, i.e., rather smooth vs. irregular and radial surface structures (Figs 2 and 7) may provide insights into its formation process. Lava domes extruded on flat surfaces grow axi-symmetrically during their early stages and spread laterally and inflate vertically in an endogenous regime of growth that represents a thermomechanical continuum^26^. Inflation of this endogenous lava dome may cause radially developed fractures at its surface, which could explain the irregular and rift-valley-like surface manifestations of the Kikai dome (Figs 2 and 7). This regime was then interrupted by lava flow that is erupted directly at the surface, a regime known as exogenous growth^26^. The western-side of the Kikai central dome with flat surfaces may thus consist of such exogenous lava flows.

### Giant lava dome magma, a remnant of supereruption?

Understanding changes in the processes of magma production and storage that take place before, during, and after the supereruption and caldera collapse has important implications for the development of a supervolcano and for the catastrophic volcanic hazards. Genesis of voluminous rhyolitic magma coming up to 32 km^3^ that formed the Kikai central dome after a rather short interval of <7,300 years from the caldera-forming supereruption should provide a key to comprehending the evolution of magma-plumbing system in the supereruption cycle of this caldera. Two contradictory scenarios would arise. One is that this voluminous post-caldera magma is the remnant of the 7.3-ka climactic eruption and the other that the magma taps a re-established reservoir beneath this supervolcano. To assess these, chemical compositions of Kikai central dome rhyolites are compared with those of syn- and post-caldera ejecta.

Essential pumices in both plinian fall deposits and ignimbrites during the 7.3-ka Akahoya supereruption exhibit a wide range of compositions and form a well-define chemical trend, which is slightly but clearly distinct from that of post-caldera ejecta (Fig. 5). Importantly, compositions of rhyolites consisting of the giant central dome lie astride the chemical trend of post-caldera ejecta, leading to the conclusion that this voluminous magma is not supplied from a melt-dominant zone within a large melt-crystal mush, or a magma reservoir, that extruded ~500-km^3^ magma during the 7.3-ka supereruption. In Long Valley caldera, 90–100 km^3^ of post-caldera rhyolite erupted after the 767-ka supereruption of 650 km^3^ of rhyolite magma as the Bishop Tuff, and exhibits chemical ranges lying within those of the zoned Bishop Tuff for most elements and some isotopes^23^. Further high-resolution geochemical examination for post-caldera stage rocks and geophysical imaging focusing on the region of magma storage in Kikai caldera are needed in order to reveal the evolution of the whole caldera cycle.

## Methods

### Remotely operated vehicle (ROV) observation

The observations of the seabed were conducted with ROV ‘SHINDAI-2K’ (Kowa Corporation), which is operated by the control unit onboard the T/S Fukae Maru with monitoring the video camera images and ROV information. The ROV is equipped with several instruments including 2 K video cameras, LED lights for illumination, laser beams for measuring the length of an object in a video camera image and an omnidirectional sonar for detection of surrounding objects. The pictures shown in Figs 4b,c and 6b were snapshots from the videos shot during ROV diving. The ROV has neutral weight in seawater and is connected to a sinker to stabilize position of ROV with a 50-m tether cable. The ROV can move around the sinker within 50 m near the seafloor. The ROV attitude and location are determined by magnetic and acceleration sensors and a depth-meter equipped with the ROV and by Ultra Short Base Line communication between the T/S Fukae Maru and the ROV using acoustic transponders.

### Multibeam and water column data acquisition and processing

Multibeam and water column data acquisition was carried out with a 40–100 kHz EM712 multibeam sonar system (Kongsberg). To ensure a sub-metric error on the beam positioning, the system was interfaced with Differential Global Positioning System and Motion Reference Unit (Kongsberg) for attitude determination and motion compensation. The acoustic ping has 40–100 Hz frequencies, 140° for the whole opening of the transmitted pulse, and 400 beams or 800 beams by dual swath. The *in-situ* water sound speed was provided for a proper beam steering by two ways; continuous sound velocity probes were carried out by an on-keel station, located near the multibeam transducers, and the sound-speed profiles in the water column were acquired by XCTD profiling every leg of the cruise (approximately once a week). Data were processed with the Seafloor Information System (SIS) software. Noise reduction due to accidental-instrumental spikes and poor quality beam exclusion were carried out by mean of swath editing and de-spiking tools. The obtained bathymetry covers about 510 km^2^ (690 m in the maximum depth) around the Kikai caldera with a resolution of 1 arc second.

### Seismic data acquisition and processing

High-resolution seismic reflection data were acquired with a single air gun, (mini GI gun, Sercel) and a hydrophone array streamer (mini-streamer, Teledyne) employed on the T/S Fukae Maru. Seismic pulses were generated by the mini GI gun configured of a 30 in.^3^ generator and a 30 in.^3^ injector that suppresses the undesired bubble oscillation. Seismic reflections were received by the mini-streamer which consists of 6 channel hydrophones with a 6.25 m group interval. The air gun and the hydrophone array streamer were towed at depths of 2 and 3 m below sea surface, respectively to maintain high frequency contents in the seismic signals. The received reflection signals were recorded at an 1-msec. sampling rate by the DAQlink III seismograph (Seismic Source). The vessel run at an average speed of 4.5 knot with the air gun firing every 8 seconds. The seismic data contain a wide frequency range of 50–260 Hz, which can identify highly resolved seismic reflectors. The acquired data was processed with static correction, band-pass filter, amplitude recovery, constant velocity stack with 12.5 m bin, and stolt migration. The data are also visualized with an Automatic Gain Control.

### Rock sampling and analyses

We collected rock samples on the lava dome by dredging from the T/S Fukae Maru and by diving and hitting with a hammer. Rock samples were cut into slabs, repeatedly boiled in distilled water for removing brine contamination, coarsely crushed and then powdered in an alumina mil. Major element compositions were obtained by a standard XRF technique with fused glass beads.

### Data availability statement

At the present stage we have no supplementary information accompanying this manuscript. However, we are happy with publishing such information if necessary.
